# Role of ROS in Aβ42 mediated cell surface P-selectin expression and actin polymerization

**DOI:** 10.1186/1750-1326-8-S1-P44

**Published:** 2013-09-13

**Authors:** Andrey Tsoy, Sholpan Askarova, Tamara Shalakhmetova, Bauyrzhan Umbayev, Shalkar Adambekov, Zhaksybay Zhumadilov

**Affiliations:** 1 Center for life sciences, Nazarbaev University, Astana, Kazakhstan; 2 Department of Biodiversity and Bioresources, Al-Farabi Kazakh National University, Almaty, Kazakhstan

## Background

Blood Brain barrier (BBB) dysfunction plays an important role in the onset and progression of Alzheimer’s disease (AD). In the AD brains an increased deposition of Aβ in the cerebral vasculature has been found to correlate with increased transmigration of blood-born inflammatory cells and neurovascular inflammation. Transmigration of leukocytes into brain parenchyma is a sequential process starting with primary capture to the endothelium and rolling adhesion mediated by tethering on selectins and selectin ligands. P-selectin is a type I transmembrane cell adhesion molecule which is stored in cytoplasmic Weibel-Palade bodies (WPb) and can be mobilized on the endothelial cell surface within minutes upon exposure to different proinflammatory agents; then it’s rapidly cleared through endocytosis in 30 min. It has become evident that Aβ activates cerebral endothelial cells (CECs) and induces mobilization of P-selectin to the cell surface. However, expression mechanisms of this receptor on the surface of brain endothelial cells under administration with Aβ42 remain unclear. Since P-selectin is stored in WPb, and there is an evidence of active role of ROS (reactive oxygen species) and actin filaments in the different stages of WPb exocytosis, in this study we examined the dynamic of P-selectin expression on the endothelial cell surface activated by Aβ42 in relation to ROS and actin polymerization.

## Materials and methods

In this study we used immortalized CECs (bEnd3) as following: control; cells incubated with Aβ42 for 10, 30 and 60 min; cells incubated with 30 mM of antioxidant N-acetylcysteine (NAC) for 1 hr; cells pretreated with NAC followed by Aβ42 exposure. Fluorescent microscopy of anti-P-selectin, dihydroethidium (DHE), and Oregon green phalloidin was used to quantify the surface P-selectin expression, ROS production, and actin polymerization.

## Results

We have observed that Aβ42 promoted ROS production up to 34.5%, 61%, 83% and increased P-selectin fluorescence up to 28%, 46%, 54% after 10 min, 30 min and 60 min of treatment respectively. In contrast, pre incubation of cells with NAC prevented the superoxide production and attenuated Aβ42 induced P-selectin activation. At the same time, NAC alone significantly reduced DHE fluorescence, but did not affect expression of the adhesion receptor. QIM data also demonstrated that Aβ42 promoted actin polymerization in cells, while pretreatment with NAC was able to suppress Aβ-induced actin polymerization and cytoskeletal reorganization in bEND3 cells.

## Conclusions

The results of our study have indicated that Aβ42 induces expression of P-selectin on the surface of bEnd3 cells and promotes actin polymerization in a time dependent manner, and all these events correlate with ROS generation. The rapid, posttranslational cell signaling response, mediated by ROS, may well represent an important physiological trigger of the micro vascular inflammatory response in AD and requires further investigations.

